# Are simulated patients effective in facilitating development of clinical competence for healthcare students? A scoping review

**DOI:** 10.1186/s41077-016-0006-1

**Published:** 2016-02-16

**Authors:** Brett Williams, Jane Jee Yeon Song

**Affiliations:** Department of Community Emergency Health & Paramedic Practice, Monash University, Peninsula Campus, McMahons Road, PO Box 527, 3199 Frankston, VIC Australia

**Keywords:** Simulated patients, Standardized patients, Clinical competence, Healthcare students

## Abstract

**Background:**

The need to evaluate the effectiveness of SPs in improving clinical competence has attracted a heightened interest across the healthcare professions, with some prevailing gaps in their evidence. Using a scoping review approach, this study aims to provide an overview on the effectiveness of SPs in facilitating the development of clinical competence for healthcare students.

**Methods:**

This scoping review applied the first five out of the six-stage methodological framework developed by Levac et al. (Implementation Science 5:69), as follows: 1) Identify the research question; 2) identify relevant studies; 3) study selection; 4) charting the data; and 5) collating, summarising and reporting the results. The search was performed on four databases, including Medline, EMBASE, CINAHL and Scopus.

**Results:**

A total of 33 articles were included in this study (out of 968 identified), comprising of 20 cross-sectional studies, eight randomised controlled trials and five longitudinal studies. The studies were examined and categorised for further discussion in the three domains of clinical competence; technical, non-technical and cognitive skills. Overall, 24 out of 33 studies showed effectiveness of SPs in facilitating students’ clinical competence.

**Conclusion:**

This scoping review serves to provide guidance for future healthcare education development, by illustrating the effectiveness of SPs in improving students’ clinical competence as evidenced in the literature. In doing so, it highlights the potential of SPs in facilitating students’ acquisition of the necessary skills for clinical practice.

## Background

With the recent advances in healthcare, there has been a growing focus on education of students and trainees towards adopting a more patient-centred approach. The traditional concept of paternalism in healthcare [1], where doctors hold the power over patients’ welfare and management, has been increasingly outdated by a more balanced view of shared decision-making [2, 3]. Easier accessibility to information through improved technology and media has also contributed to this shift in the relationship between professional and patient [4]. Consequently, it has become more important for healthcare professionals to listen and incorporate patients’ viewpoints and address their needs, which can be achieved through interaction and valuable feedback from patients. In doing so, it allows healthcare professionals to enhance their diagnostic, communication and professional skills [5].

However, with time constraints and unpredictable nature of real-life encounters, it is usually not possible to accomplish such ideal situations for appropriate clinical teaching involving feedback from patients [6]. Healthcare professionals or students are therefore likely to benefit from structured simulated learning where they can develop the necessary skills for safe practice before they are faced with complex and unpredictable encounters in the real world. One such simulation-based learning approach is simulated patients (SPs), also known as SP methodology.

Simulation-based education involving SPs has been designed to contribute to solving this challenge, with its role progressively growing in the field of healthcare professional education [7, 8]. The SP methodology offers the benefit of providing students with a safe environment that is readily available, which can be adapted to specific learning purposes [9]. For example, training sessions working with SPs can be arranged at suitable times and places as required, while real-patient encounters are generally limited to general practice or hospital settings. SP sessions also allow mistakes and interruptions to be made for student feedback as part of the teaching process.

David Gaba, an innovator in modern healthcare simulation, defines simulation as ‘a technique to replace or amplify real experiences with guided experiences…that evoke or replicate aspects of the real world in a fully interactive fashion’ [10]. This definition essentially encompasses the role of a SP in healthcare education, which is to provide a high fidelity learning environment that realistically replicates a patient encounter, by portraying a patient in a predetermined clinical scenario [11]. SPs are trained to follow a script to reproduce a particular problem or symptoms, and are given a set of guidelines to follow for certain responses. They also provide specific patient-centred feedback that many healthcare professionals and students require in order to further enhance their learning and skills [12].

The terms ‘simulated’ and ‘standardised’ patients are often used interchangeably within the literature, although there are subtle differences. For example, the term ‘simulated patients’ refers to persons with generic role of portraying a patient in a clinical scenario, often used in training for educational purposes. In contrast, the term ‘standardisation’ confers a stricter criterion, where SPs must perform consistently according to the predetermined learning purposes, often used in high stakes assessments for objectivity [13]. In addition, there are some geographical differences, with the term ‘simulated patient’ generally used in Australia and the United Kingdom, while the term ‘standardized patient’ is more common in Canada and North America [14]. For the purpose of this review, the two terms will be used interchangeably, with common abbreviation of ‘SP’.

With the increasing contribution by SPs to healthcare professional education, the need to evaluate their effectiveness has consequently attracted a heightened interest across the healthcare professions [15]. Indeed, the value of health professional encounters with SPs has been well documented in literature [16–18]. Its impact is also demonstrated by many Australian Universities and other training providers individually investing time and money in recruiting and training SPs. For example, Gippsland Medical School, Australia had 45 SPs on register in 2011, with focused training programs funded by the government for teaching and assessment purposes [13]. Additionally, the Victorian Simulated Patient Network showed membership of 437 in 2014, with SP programs implemented in major Australian Universities including The University of Melbourne, University of Tasmania, University of Queensland and Monash University [19].

As a ‘bridge’ to the real-world experience, SPs provide a safe environment where students can practise and refine their clinical skills [20]. In a study examining students’ views on working with SPs, encounters with SPs were found to be more beneficial in developing communication skills and self-confidence compared to encounters with real patients [21]. Their role is further extended by a ‘hybrid model’, which is designed to allow technical skills such as wound suturing and catheter insertion to be performed on inanimate models attached to SPs. It provides opportunities for students to practise procedural skills while enhancing their interpersonal skills in a convincing learning environment [22]. In one study, such models were evaluated to be significantly more effective in improving students’ clinical competence than the traditional simulation-based training with mannequins [23].

However, some gaps still continue to exist in terms of the evidence for SPs’ effectiveness and means of evaluating the role of SP methodology in professional education, with some identifiable challenges that limit their usefulness. For example, although a recent systematic review concluded practice-oriented strategies to be effective in physician-training programs, it did not demonstrate the significance of SPs as an individual strategy [24]. Furthermore, while the nature of SPs requires strict criteria and scripts to be followed in order to portray a predetermined clinical scenario with fidelity and objectivity, the authenticity of such experience may be questioned, as some elements of clinical performance represented by SPs are unlikely to be considered in such detail in real patients [25].

This study aims to explore the role of SPs in healthcare education using a scoping review approach, assessing their effectiveness and demonstrating the ways in which they function to facilitate development of the required skills amongst healthcare students. The methodology of scoping review was considered appropriate for this study, as it allows more comprehensive and broader objectives through the use of wider-ranging literature applicable to a particular topic [26].

## Methods

Scoping reviews allow mapping of the key concepts and the evidence supporting a research domain of interest. In doing so, they establish the current state of knowledge while identifying gaps in the existing literature [26, 27]. Compared to systematic reviews with strict criteria for study inclusion, scoping reviews incorporate a wider range of research materials that are both peer-reviewed and non-peer-reviewed in order to provide a broader understanding of the topic, which can then be narrowed down to focus on the specific research question [26, 28].

This scoping review applied the first five out of the six-stage methodological framework developed by Levac et al. [29] as follows:

1. Identify the research question
2. Identify relevant studies
3. Study selection
4. Charting the data
5. Collating, summarising and reporting the results
6. Consultation (stage 6 is not included in this study).

In the original methodology for scoping reviews formulated by Arksey and O’Malley in 2005, the sixth stage consultation was recommended to be optional to provide additional perspectives on the topic and further validate the findings [26]. This sixth stage was however made compulsory in the methodology devised by Levac et al. [29]. Unfortunately, decisions have been made by the authors to exclude this sixth stage in this review due to limited resources.

### Identify the research question

This scoping review was conducted to answer the following research question: Are simulated patients effective in facilitating the development of clinical competence for healthcare students? This question allowed the examination of materials from an extensive domain within the healthcare setting, while focusing on the impact of SPs on clinical competence of students.

### Identify relevant studies

Table 1 outlines the strategy used to perform the search. Three main search terms were identified from the research question: simulated patients, clinical competence and healthcare students. Both terms ‘simulated patients’ and ‘standardized patients’ were accepted as they are used interchangeably within the literature [14].

**Table 1:** Search strategy

1. Simulated adj patient* 2. Standardi#ed adj patient* 3. 1 or 2 4. Exp clinical competence 5. Clinical adj skill* 6. Clinical adj performance* 7. Technical adj skill* 8. Procedural adj skill* 9. Examination adj skill* 10. Non-technical adj skill* 11. Communication adj skill* 12. Teamwork* adj skill* 13. Cognitive adj skill* 14. Decision adj making adj skill* 15. Clinical adj reasoning adj skill* 16. 4 or 5 or 6 or 7 or 8 or 9 or 10 or 11 or 12 or 13 or 14 or 15 17. Exp student, health occupations 18. Healthcare adj student* 19. Health adj occupation* adj student* 20. Occupation* adj student* adj health 21. Student* adj health adj occupation* 22. 17 or 18 or 19 or 20 or 21 23. 3 and 16 and 22	

*Used for truncation during database searches

The list of terms searched for the concept of ‘clinical competence’ was derived from the definition devised by the National Health Service (NHS) [30], which categorises clinical skills into three main components:

1. Technical skills, including procedural and examination skills
2. Non-technical skills, including communication and teamwork skills
3. Cognitive skills, including decision making and clinical reasoning skills.

The term ‘healthcare students’ was used to include students from all healthcare disciplines.

**Fig. 1:**
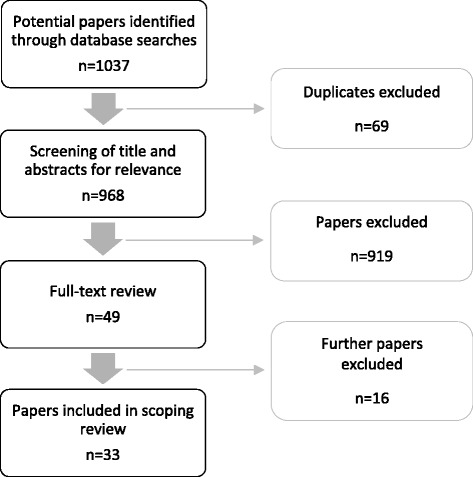
Results of search strategy and process of study selection

A total of four databases were searched, including Medline, EMBASE, CINAHL and Scopus. Grey literature sites were also searched, including Grey Literature Report: http://www.greylit.org/ and Australian Open Access Support Group: http://aoasg.org.au/. Initial search of the electronic databases resulted in 968 articles, excluding duplicates. Screening of the titles and abstracts undertaken by BW and JS for relevance rendered 49 articles according to the inclusion and exclusion criteria as outlined in the next section, from which 16 were excluded after the full text analysis as they did not meet the criteria. Finally 33 were selected for inclusion in this scoping review. The results of the search strategy are shown in Fig. 1.

### Study selection

The selection of articles for study inclusion was based on the following criteria:

1. Involvement of SPs as part of a teaching activity or educational intervention

2. Objective measurement of clinical competence as part of the outcome of both summative and formative assessments

3. Undergraduate students from healthcare discipline as the study population.

Articles that only measured perception or attitude of students, such as self-reported ratings, were excluded. Other exclusion criteria included having non-students as the only study population, and non-English publications.

The two authors (BW and JS) reviewed the titles and abstracts of the articles retrieved from the database search. Based on the agreement of the authors, the relevant articles were subsequently subjected to full-text analysis (BW and JS) to be chosen for this scoping review.

### Charting the data

The ‘narrative review’ approach by Pawson [31], adopted in this review, aims to include a broader context of the results to provide readers with a better understanding. It necessitates thoughtful decisions regarding what information is to be included and how the results should be compared [31]. Using this approach, the key information from the chosen articles were charted according to a common analytical framework [26] consisting of main headings as follows: study location; study type; cohort size; cohort discipline; intervention; comparison; outcome assessed; and main findings including statistical results.

## Results

### Collating, summarising and reporting the results

A total of 33 articles were included, comprising 20 cross-sectional studies, 8 randomised controlled trials and 5 longitudinal studies, published between 1997 and 2013.

Results are presented below and a summary is outlined in Table 2. The full list of references concerning the accepted studies is given in Appendix 1.

### Study location

Most of the studies were conducted in the United States (20 studies), while the rest was undertaken in other countries including Canada (4 studies), Belgium (2 studies), Germany (2 studies), Belgium (1 study), United Kingdom (1 study), Turkey (1 study), Taiwan (1 study) and India (1 study).

### Professional area(s) under assessment

The majority of the studies examined medical students (25 studies), with students from nursing (4 studies), dentistry (2 studies), pharmacy (1 study), occupational therapy and physiotherapy (1 study) being also studied.

## Discussion

Clinical competence is a fundamental quality of healthcare professionals. As such, a substantial focus has been placed over the years on ways to facilitate healthcare students in developing optimal level of clinical competence, with SPs becoming increasingly used as an educational tool. This scoping review examined 33 articles that assessed the effectiveness of working with SPs in this regard, under the three main domains of clinical competence. These will now be discussed.

### Technical skills

Traditionally, technical aspects of clinical competence such as physical examination skills have been taught using a didactic approach, often involving textbooks and lecture notes limiting student’s opportunities to perform and practise the necessary skills [3, 32]. In order to overcome these limitations and move towards a more self-directed and interactive learning, SPs have been introduced over the past 50 years [33] in the field of healthcare education.

Within the included articles that examined the impact of SPs on students’ technical skill development, the majority (8 out of 11 studies) supported the view that SPs are effective in improving students’ examination skills. SPs were effective at reinforcing knowledge attained from the traditional didactic learning through actual performing of manoeuvres on a real person. SPs could also provide direct feedback, which allowed students to reflect on their performance and improve on areas of weakness.

For example, in a study by Safeieh [34] involving medical students, a single training session with SPs on breast and abdominal examination techniques in addition to a standard textbook teaching resulted in a significantly higher performance scores than standard textbook teaching only (*p* = 0.002 and <0.001 on breast and abdominal examinations respectively). Moreover, calculation of pre- and post-test scores showed a greater improvement of scores in students who received a training session with SPs as compared to those who did not (*p* = 0.036 and <0.001 on breast and abdominal examinations respectively).

This positive impact of working with SPs is further shown in a study by [35] which found that an additional training session with a SP resulted in a significantly higher performance in clinical breast examination among third-year medical students, with a *p* = 0.04.

However, there has been some concern that these positive effects of SP training sessions on medical students’ clinical competence may only be short-term, as most published data examined outcomes that were measured soon after the intervention [36, 37]. Safdieh et al. [34] attempted to address this concern by analysing student performance 2 years after the intervention. The trial showed that medical students who received neurological examination session by SPs as part of their second-year curriculum performed better than those who did not receive the session by SPs, as measured in an objective-structured clinical exercise at the end of their fourth-year, with a *p* < 0.001.

These studies confirm the important role of SPs in providing students with opportunities to develop and enhance their technical skills such as examination skills, which can best be done through practice. SPs also seem to have a long-term benefit in technical skills acquisition for medical students.

### Non-technical skills

Effective communication skills play an integral part of successful interaction between healthcare professionals and patients [38]. SPs provide an opportunity to practise such skills where students are able to interact and communicate with patients, while learning and developing their interpersonal skills.

Analysis of the included articles reflected an emphasis on this domain of clinical competence in particular, with a majority of them focusing on the effectiveness of SPs in students’ development of non-technical skills, such as communication or interpersonal skills. Overall, 16 out of 22 concluded that programs or training sessions by SPs result in better performance of communication skills in students than without SP involvement.

The above referred pilot study by Bachmann et al. [38] showed that undergraduate medical students who underwent a brief two-hour communication skills training performed better in a primary care communication examination than students who had no training (*p* = 0.02). The study also commented on its feasibility of such intervention for other healthcare professions.

The benefit of working with SPs in improving students’ communication competence seem to be further enhanced by ‘hybrid simulation’, which is essentially an integration of SPs and mannequins [39]. It allows students to verbally interact with the SP while performing practical manoeuvres on a part of the mannequin attached to the patient, thereby developing their communication competence while practising technical skills at the same time.

The same study [39] found that training fourth-year medical students with hybrid simulation resulted in higher communication scores than training in small-group tutorials, with a *p* = 0.01, supporting the potential of SPs as an effective additional tool in communication skill development.

However, a minority (6 out of 22 studies) failed to show effectiveness of SPs in facilitating non-technical skill development. For instance, a randomised controlled study by [40] involving 129 nursing students compared the effect of adding SPs to didactic lectures-only on students’ communication skills. Analysis of post-encounter SP checklist scores, which objectively measured students’ performance on communication and interpersonal skills, showed no significant difference between the two groups (*p* = 0.238).

Despite variability in the results, the majority of studies (16 out of 22) supported working with SPs in helping students develop communication and interpersonal skills. This was evidenced by favourable outcomes of student performances in majority of the cases, indicating the advisability of working with SPs in healthcare education for students’ non-technical skills development.

### Cognitive skills

The cognitive aspect of clinical competence may include skills such as decision-making and clinical reasoning [30]. History-taking is also one of the important skills under this domain, as obtaining sufficient information from effective history-taking allows healthcare professionals to make correct diagnosis and take appropriate management actions [41].

The effectiveness of SPs in assisting students’ development of such skills was highlighted in 8 out of 11 studies.

For example, Haist et al. [42] reviewed the impact of a four-hour workshop working with SPs on third year medical students in improving their clinical diagnosis skills, specifically on the topic of domestic violence. Students’ performance on checklist examinations was measured at four and 27 weeks after the workshop, and was compared to those who did not participate in the workshop. Results showed significant difference in the scores between the two groups, both at four and 27 weeks, with *p* = 0.002 and *p* = 0.01 at respective times, favouring the students who had participated in the workshop working with SPs.

Overall, SPs were assessed to be effective in facilitating students’ cognitive skills. Both short-term and long-term benefits of SPs were seen, demonstrating their potential to be used as an effective means of education.

### Limitations and future research

While scoping reviews offer a number of advantages over other methodological approaches in literature reviews, including a relatively shorter research process and a broader range of data that can be examined, there are certain limitations to be addressed, which are discussed below [28].

Firstly in this scoping review, the quality of evidence in the research articles was not assessed or appraised, as opposed to in systematic reviews. This hinders the measurement of validity and generalisability of such findings from these studies. Secondly, as a considerable amount of data was extracted in the preliminary research, specific and focused inclusion and exclusion criteria had to be applied, in order to obtain articles that met the objective of this scoping review. For example, only the terms ‘simulated patient’ and ‘standardized patient’ were used for the search, while literature describes other terms that can be used as alternatives such as ‘trained patient’ or ‘actor patient’ [14]. This may have missed some literature that could have been relevant in this review. Furthermore, only the studies published in English were included, limiting the scope of literature to be considered.

Nevertheless, this scoping review provides a valuable overview of the current literature regarding the effectiveness of SPs in healthcare education. Although most of the data identified in this review (24 out of 33 studies) seems to favour working with SPs in facilitating students’ clinical competence, there was some variability of findings as indicated by 9 studies that failed to show effectiveness of SPs. One should consider variable factors that may have contributed to such findings, such as small sample size, and carefully review the methodology used in the studies and the implications of statistical significance.

With the nature of scoping reviews which incorporates a wide ranging research data, a more rigorous search using systematic approach will help yield a more definitive conclusion on the topic. It may also be interesting to focus on quantitatively comparing the effectiveness of SPs with other methods of teaching clinical skills in order to aid in future development of education for healthcare students. Furthermore, inclusion of SPs’ associated costs may be of a practical benefit, essentially when universities must decide on working with SPs at a time when resources are scarce, thereby providing useful information for future directions in healthcare education.

## Conclusion

With the emergence of SPs in healthcare education, there have been questions of their effectiveness as an educational tool for students. This scoping review aimed to provide with an overview of the available research data on the effectiveness of SPs in facilitating healthcare students’ clinical competence.

The majority of studies, namely 24 out of 33 studies, supported the use of SP methodology in healthcare education, showing evidence for their effectiveness in students’ development of clinical competence.

In this way, this scoping review serves to provide guidance for future healthcare education development, by highlighting the effectiveness of SPs in facilitating students in acquiring the necessary skills for clinical practice. Furthermore, it bears important implications for healthcare professions, as incorporation of SPs into healthcare education has the potential to not only improve consistency in skill practice, but also reduce the cost of employing clinicians when such resource may be scarce. Working with SPs may also allow further professionalization of lay-educators in this field, and provide positive contribution to development of inter-professional education across different healthcare sectors.

## Appendix

**Table 2:** Studies selected for inclusion

#*	Country	Study type	Cohort size	Cohort discipline	Intervention	Comparison	Outcome assessed	Main findings
Cognitive skills								
1	U.S.	Cross-sectional	85	Medicine	4-h workshop using SPs	Non-participants	Diagnosing domestic violence	Better performance
2	U.S.	Cross-sectional	263	Medicine	Curriculum involving SPs	Non-participants	Pain assessment and management skills	Better performance
3	Belgium	RCT (3-factor)	196	Medicine	Training session with SPs	Training with physicians or online training	Cognitive component of consultation skills	No improvement (cf. improvement in comparison group)
4	Canada	Cross-sectional	72	Occupational therapy & physiotherapy	Interaction with SPs	Watching videotapes	Treatment planning	Worse performance
Non-technical skills								
5	U.S.	Longitudinal	143	Dentistry	Training session with SPs	-	Interpersonal communication skills	Improvement of performance
6	Turkey	Longitudinal	146	Medicine	Training session involving SPs	-	Breaking bad news	Improvement of performance
7	U.S.	Longitudinal	45	Medicine	2-h curriculum by SPs	-	Brief motivational interviewing skills	Improvement of performance
8	U.S.	Longitudinal	127	Pharmacy	Lecture-laboratory course with SPs	-	Communication skills	Improvement of performance
9	Germany	Cross-sectional	286	Medicine	2-h communication skills training session with SPs	Non-participants	Communication skills	Better performance
10	Germany	Cross-sectional	103	Medicine	Training session with SPs	Non-participants	Communication skills	Better performance
11	U.S.	Cross-sectional	38	Medicine	1-h session with SP	Non-participants	Breaking bad news	Better performance
12	U.S.	RCT	186	Medicine	4-h workshop using SPs	Non-participants	Interviewing and counselling skills	Better performance
13	U.S.	Cross-sectional	106	Medicine	Educational session involving SPs	Non-participants	Communication skills	Better performance
14	India	RCT	60	Dentistry	Training course involving SPs	Non-participants	Communication skills	Better performance
15	U.K.	RCT	24	Medicine	Hybrid simulation session with SPs	Small group tutorial	Communication skills	Better performance
16	Canada	RCT	32	Medicine	SP-session with feedback	SP-session without feedback	Communication skills	Better performance
17	U.S.	RCT	129	Nursing	Additional teaching sessions with SPs	Didactic lectures only	Interpersonal and therapeutic communication skills	No difference
18	Taiwan	RCT	26	Nursing	Instructional class with additional SP feedback and group discussion	Instructional class only	Communication skills	No difference
19	U.S.	RCT	93	Medicine	Role-play session with SPs	Session with student colleagues	Motivational interviewing skills	No difference
20	U.S.	Cross-sectional	72	Medicine	Practising with SPs	Role-playing with student colleagues	Smoking-cessation counselling skills	No difference
Cognitive and non-technical skills								
21	U.S.	Cross-sectional	85	Medicine	4-h workshop using SPs	Non-participants	Sexual history and HIV counselling skills	Better performance
22	U.S.	Cross-sectional	183	Medicine	Workshop involving SPs	Non-participants	History taking and counselling skills	Better performance
Technical skills								
23	U.S.	Cross-sectional	95	Medicine	Additional training session with SP	No additional session	Breast examination skills	Better performance
24	Belgium	Cross-sectional	102	Medicine	Training session with SP in a new curriculum	Non-participants	Urological and gynaecological examination skills	Better performance
25	U.S.	Cross-sectional	89	Medicine	Additional teaching with SPs	Traditional teaching only	Breast and abdominal examination skills	Better performance
26	U.S.	Cross-sectional	187	Medicine	Curriculum with additional SP session	Standard curriculum only	Neurological examination skills	Better performance
27	U.S.	Cross-sectional	244	Medicine	Teaching session with SPs	Breast-palpation simulator	Breast examination skills	Worse performance
Technical and cognitive skills								
28	Canada	Cross-sectional	108	Nursing	Practice with SPs	Practice on student colleagues	History taking and examination skills	Better performance
29	U.S.	Cross-sectional	204	Medicine	Lecture with additional SP exercises	Lecture only	History taking and examination skills	Better performance
Technical and non-technical skills								
30	Canada	Cross-sectional	82	Medicine	2-h teaching session by SP	Physician-led teaching	Interpersonal and musculoskeletal examination skills	No difference
Cognitive, technical and non-technical skills								
31	U.S.	Longitudinal	622	Medicine	Curriculum involving SPs	-	History taking, communication and examination skills	Improvement of performance
32	U.S.	Cross-sectional	170	Medicine	Training session with SPs	Non-participants	Information gathering, communication and examination skills	Better performance
33	Canada	Cross-sectional	44	Nursing	Training session with SPs	Human simulators or community volunteers	History taking, communication and examination skills	Worse performance

*Authors corresponding to # in the Table 2

1. Haist et al. (2003)

2. Stevens et al. (2010)

3. Aper et al. (2012)

4. Liu et al. (1997)

5. Broder et al. (2006)

6. Dikici et al. (2009)

7. Martino et al. (2007)

8. Rickles et al. (2009)

9. Bachmann et al. (2013)

10. Bosse et al. (2012)

11. Colletti et al. (2001)

12. Feddock et al. (2009)

13. Lorin et al. (2006)

14. Sangappa et al. (2013)

15. Siassakos et al. (2010)

16. Moulton et al. (2009)

17. Becker et al. (2006)

18. Lin et al. (2013)

19. Mounsey et al. (2006)

20. Papadakis et al. (2009)

21. Haist et al. (2004)

22. Hoellein et al. (2009)

23. Barrett et al. (2002)

24. Hendrickx et al. (2009)

25. Sachdeva et al. (1997)

26. Safdieh et al. (2011)

27. Schubart et al. (2012)

28. Bornais et al. (2012)

29. Fletcher et al. (2004)

30. Oswald et al. (2011)

31. Supiano et al. (2007)

32. Sutin et al. (2011)

33. Luctkar-Flude et al. (2012)
